# The Austrian Osteopathic Practitioners Estimates and RAtes (OPERA): A cross-sectional survey

**DOI:** 10.1371/journal.pone.0278041

**Published:** 2022-11-28

**Authors:** Patrick L. S. van Dun, Lorenzo Arcuri, Johan Verbeeck, Jorge E. Esteves, Francesco Cerritelli

**Affiliations:** 1 Foundation COME Collaboration, Pescara, Italy; 2 Belgium National Centre, Foundation COME Collaboration, Mechelen, Belgium; 3 Independent Statistical Consultant, Blaasveld, Belgium; Public Library of Science, UNITED STATES

## Abstract

**Introduction:**

Since the previous survey of the osteopathic profession in Austria was almost a decade ago, an update was necessary. The Osteopathic Practitioners Estimates and RAtes (OPERA) project was developed as a Europe-based survey, whereby an updated profile of the profession not only provides new data for Austria, but also allows for a clear comparison with other European countries.

**Methods:**

A voluntary, online-based, closed-ended survey was distributed across Austria in the period between April and August 2020. The original English OPERA-questionnaire, composed of 52 questions in seven sections, was formally translated in German and adapted to the Austrian situation. Recruitment was performed through social-media and an e-based campaign.

**Results:**

The survey was completed by 338 individuals, of which 239 (71%) were female, and the median age was 40–49 years. Almost all respondents had preliminary healthcare training, mainly in physiotherapy (72%). The majority of respondents were self-employed (88%) and working as sole practitioners (54%). The median number of consultations per week was 21–25 and the majority of respondents scheduled 46–60 minutes for each consultation (69%). The most commonly used diagnostic techniques were: palpation of position/structure, palpation of tenderness and visual inspection. The most commonly used treatment techniques were cranial, visceral and articulatory/mobilisation techniques. The majority of patients estimated by respondents consulted an osteopath for musculoskeletal complaints mainly localised in the lumbar and cervical region. Although the majority of respondents experience a strong osteopathic identity, only a small proportion (17%) advertise themselves exclusively as osteopaths.

**Conclusions:**

This study represents the first published document to determine the characteristics of the osteopathic practitioners in Austria using large, national data. It provides new information on where, how, and by whom osteopathic care is delivered. The information provided may contribute to the evidence used by stakeholders and policy makers for the future regulation of the profession in Austria.

## Introduction

The European Committee for Standardisation (CEN) [1] considers osteopathy or osteopathic medicine to be a primary-contact and patient-centred healthcare discipline. The terms osteopathy and osteopathic medicine are sometimes used interchangeably in some countries. Currently, osteopathy is regulated in twelve European countries (Cyprus, Denmark, Finland, France, Iceland, Italy, Liechtenstein, Luxembourg, Malta, Portugal, Switzerland, and the UK) [2]. Other countries such as Belgium and Norway have recognised osteopathy but have not yet fully regulated it [2]. The rapid growth of the profession and its global acceptance make it imperative to closely monitor demographic changes and collect data on the characteristics of osteopathic practitioners, their patients and the nature of their practice [3]. Especially in those countries where osteopathy is not regulated yet, robust data is still largely missing. Although osteopathy is not yet recognised, nor regulated, as a profession in Austria, osteopaths were already surveyed in 2003 [4] and 2011 [5] within the framework of a research dissertation, and a mixed methods study on the characteristics of the osteopathic profession in Germany, Austria and Switzerland is in progress [6].

Workforce surveys provide important information, which can be used to gain valuable insight into a profession’s ongoing and future development. The Osteopathic Practitioners, Estimates and RAtes (OPERA) project from the COME Collaboration has been developed and defined as an internationally-based survey project dedicated to profiling the osteopathic profession across Europe. This OPERA survey has already been conducted in several other European countries (Belgium, Italy, Luxembourg, Portugal and Spain) [7–11], which makes international comparison easier. The survey also includes new information about professional identity and views on the profession, questions that were not included in previous surveys conducted in Austria.

The aim of this study is to describe the current characteristics of Austrian osteopathic practitioners, work status, training, professional identity, characteristics of clinical practice with regard to consultation structure, patient profile and use of diagnostic and treatment modalities.

## Methods

This cross-sectional, online, practitioner survey followed the methods described in previous OPERA studies [7, 8]. The SUrvey Reporting GuidelinE (SURGE) [12] was used as the reporting guideline for this paper.

### Population

A voluntary, online-based, closed-ended survey was distributed across Austria in the period between April and August 2020. Eligible participants were required to fulfill the following inclusion criteria: any practitioner who works in Austria and defined him/herself as an osteopath, regardless of his/her education and academic degree(s). As the OPERA AU project was an online based survey, practitioners without internet access were indirectly excluded. Informed consent was assumed by participation in the study. The survey was approved by the Institutional Review Board of the Foundation COME Collaboration (09/2019).

### Recruitment

In order to successfully carry out the objectives of this survey, a dedicated website with general and privacy information, and FAQs was created. This website also offered a participation link for those who could not be contacted personally. Our partner in this survey was the Austrian Association of Osteopathy (Österreichische Gesellschaft für Osteopathie—OEGO), the only professional association in Austria, with 520 members in April 2020. OEGO set up an e-campaign to its members. In addition, all participating osteopathic educational institutions received a paper flyer to hang up at their location. A manual white-page search was also conducted to identify osteopaths who were not members of OEGO. To encourage participation, e-flyers were distributed to all mailing lists, during the recruitment period and during data collection, and a combined strategy of social media (Facebook, Twitter) and newsletters was implemented. Participation in the study was completely voluntary.

### Survey tool

The OPERA survey used a validated questionnaire with 52 questions, in seven sections: socio-demographics, work status and professional activities, education and life-long learning, professional identity, fee and consultation structure, patients, and osteopathic competences in diagnostics and treatment [7]. In order to enable correct interpretation of the original English version, the translation and validation into German followed the forward-backward process recommended by the WHO. It included translating and linguistic adaptation by two English-German translators-interpreters with experience in health research, blinded to previous questionnaire knowledge, and without knowing each other. The aim was to reduce the difference found in the translation, choosing, and adapting from English to the German-Austrian expressions and words that better express the osteopathic culture knowledge. A pre-pilot study was conducted with ten Austrian osteopaths to validate the questionnaire. Each osteopath was instructed to comment on the understanding of the questions/words/technical terms, and modifications were based on their observations.

The OPERA survey online platform warehouse (previously developed and improved) has been used to collect all the data that was previously encrypted and sent via the internet using an ad-hoc software named COME Survey [8]. This software runs highly secure surveys and studies containing potentially sensitive data [8]. Answers were anonymised, and IP addresses were not disclosed to the research team. The system automatically manages the link between e-mail address, study ID, and survey status, which means the research team could not identify the responses provided. Only the OPERA research team was able to access the complete and anonymous data produced by the COME Survey software.

### Privacy

The questionnaire respected the anonymity and privacy of data following the European directive 2018/1725CE of the European Parliament. De-identified and anonymised data will be stored for 5 years, available upon request, and used for further analyses and benchmarking.

### Information guidelines

Participants were asked to complete the forms by filling in the information regarding the demographics, working status and professional activities, education and life-long learning, professional identity, fee and consultation structure, patients, and osteopathic competences in diagnostics and treatment. Participants were allowed access to the survey only after receiving study information and informed consent.

### Statistical analysis

Considering a response rate of 40% (standard deviation 24) in seven osteopathic practitioner-oriented surveys [13], the population of osteopaths in Austria can be estimated to range between 580 and 1,525, an estimate that coincides with that of 1,300 of OEGO (520 OEGO-members and 780 non-members) [personal communication between PvD and OEGO].

All the information gathered was analysed and reported as aggregated data. Completed questionnaires were individually examined and no attempt was made to identify respondents. Results are presented following a descriptive analysis using frequencies and percentages for qualitative variables. R statistical programme (v.3.1.3) was used to analyse the data.

## Results

There were no drop-outs and the dataset showed that all questions had been answered by respondents and that the questionnaire had therefore been completed.

### Socio-demographic characteristics

**Fig 1:**
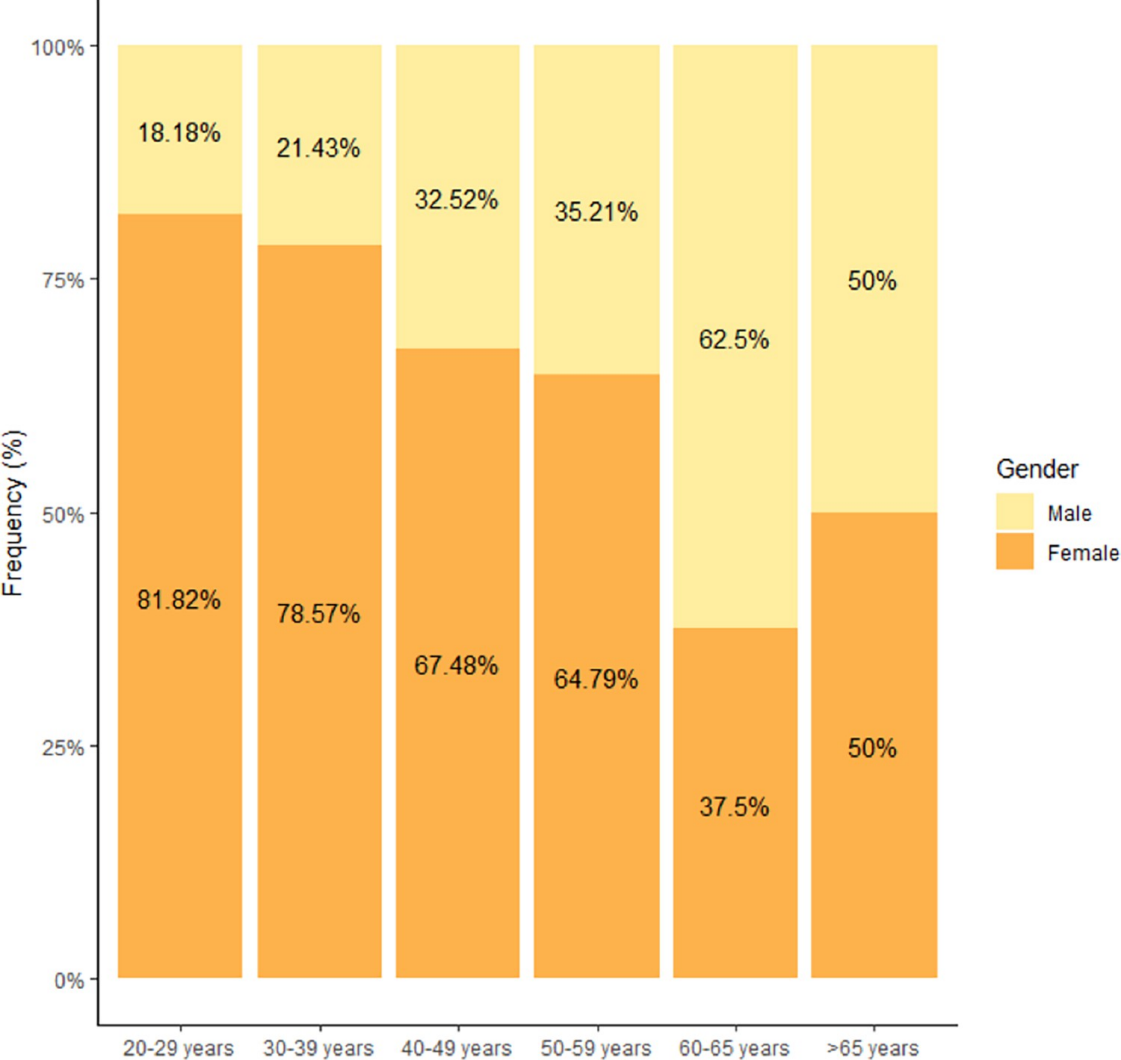
Age distribution by gender (n = 338).

A total of 338 completed the survey, of which 239 were female (70.7%). Considering an estimate of 1,300 active osteopaths in Austria, this corresponds to a response rate of 26%. There is a gender shift in the 50–59 years age category, with more males in the higher age groups (Fig 1). The majority of respondents were aged between 40–49 years (36.4%), followed by 33.1% that were between 30–39 years of age. For each respondent over 65 years of age, there were eleven respondents in the 20–29 age category.

All federal states were represented by respondents in this survey, with a regional distribution concentrated mainly in Vienna (26.0%), followed by Oberösterreich (15.4%), Niederösterreich (15.4%) and Steiermark (14.2%). Three-quarters of respondents (74.6%) are members of a professional osteopathic association (S1 Table). Table 1 shows respondents’ gender and age distribution.

**Table 1:** Gender and age distribution (n = 338).

Variable	n	%
**Gender**		
Male	239	70.7
Female	99	29.3
**Age**		
20–29	22	6.5
30–39	112	33.1
40–49	123	36.4
50–59	71	21
60–69	8	2.4
>69	2	0.6

### Work status and professional activities

**Fig 2:**
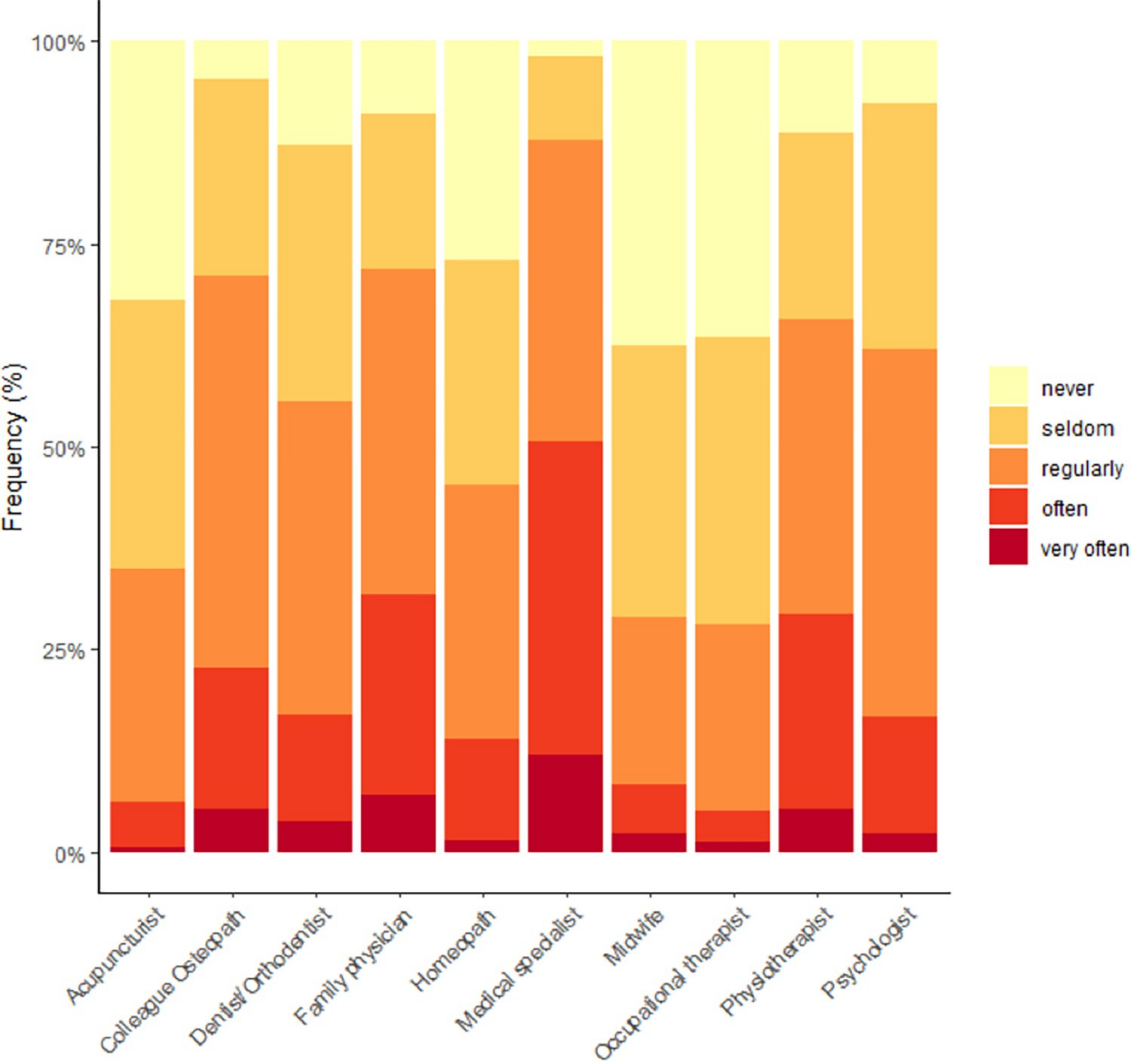
Frequency of referring patients by osteopathic practitioners to other healthcare professionals (%).

**Fig 3:**
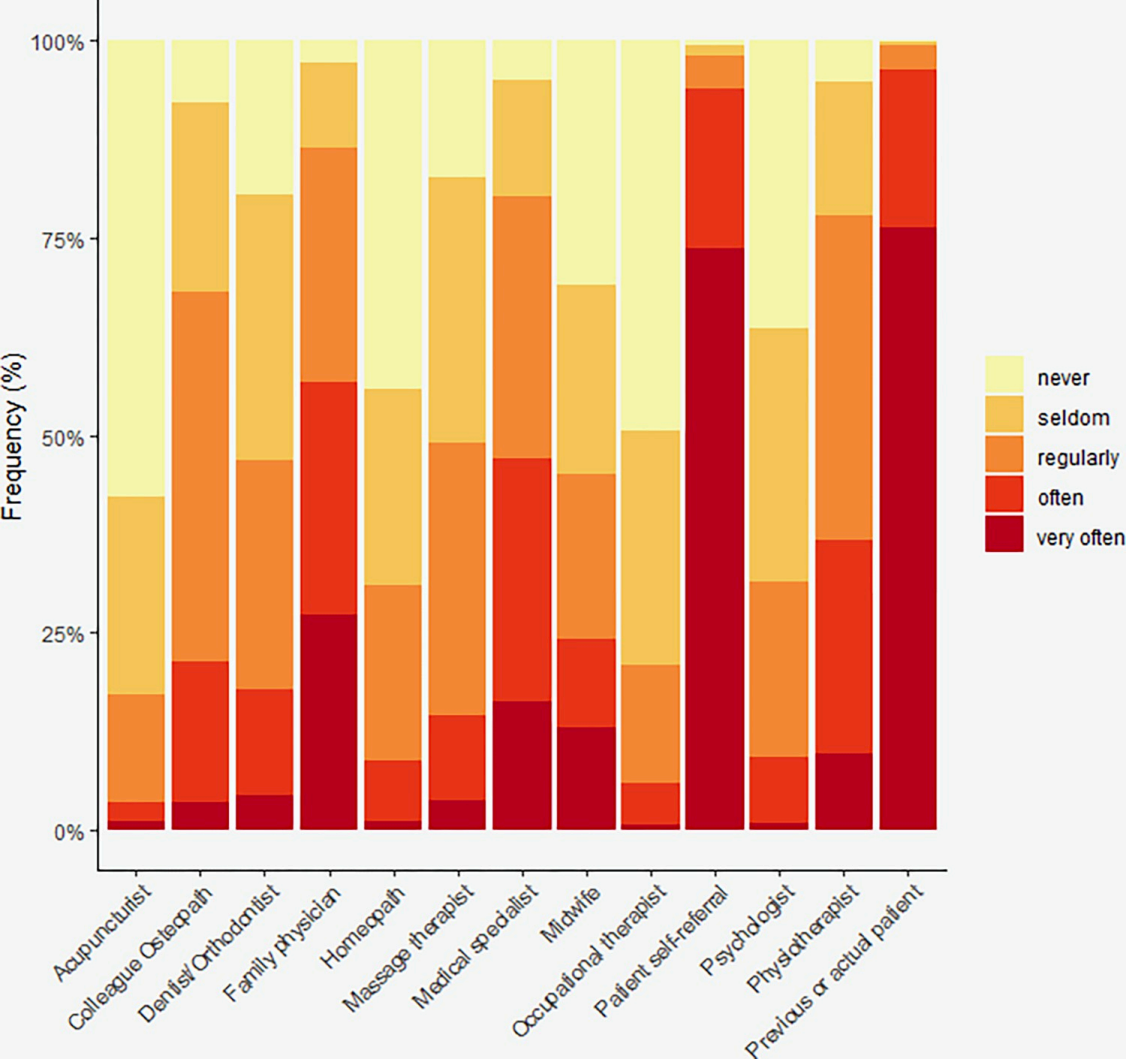
Frequency of referring patients by other healthcare professionals to osteopathic practitioners (%).

The majority of respondents were self-employed (88.2%), owner of a clinic (66.8%) of which 52.4% were the sole owners, and sole practitioners (54.1%) (Table 2). Of those who reported working as part of a team (45.9%), most did so with physiotherapists (27.9%) osteopaths (25.2%) and massage therapists (8.9%) (S1 Fig). A little more than half of the respondents (52.4%) reported having other professional activities apart from their clinical practice as osteopaths. Of these, 51.2% were also working as physiotherapists (S2 Table). Respondents reported referring patients to other healthcare professionals as shown in Fig 2. Fig 3 shows the frequency and types of referrals received by Austrian osteopaths.

**Table 2:** Work status of respondents (n = 338).

Descriptor	Variable	n	%
**Type of employment**	Self-employed	298	88.2
	Employee	6	1.8
	Both	34	10.1
**Self-employed Status**	Owner of a clinic	156	52.4
	Business partner of a clinic	99	33.2
	Associate	43	14.4
**Type of working collaboration**	Alone	183	54.1
	Only as part of a team	66	19.5
	Both alone and as part of a team	89	26.3

Thirty percent of respondents showed interest in treating specific patient groups, such as children (20.8%), pregnant women (19.7%), patients with specific pathologies (19.1%) and newborns (18%). Concerning their consultation policy, the majority of respondents informed patients about chaperone policy for minors (90.8%), consultation cancellation policy (85.8%), data handling policy (84.0%) and confidentiality policy (83.4%). Information about chaperone policy for treating intimate zones was mentioned by half of the respondents (53.9%).

### Osteopathic training and lifelong education

The most common type of osteopathic training was more than 6 years (47.6%) part-time training (97.9%) in Austria (78.7%), concluding with a master’s degree (38.8%). The second most common highest qualification at the conclusion of their osteopathic training was a Diploma in Osteopathy (DO) at 33.4%. Almost all respondents (99.76%) had a preliminary healthcare training, mainly as physiotherapists (71.5%). Of all respondents, 89.4% had participated in continuing professional development (CPD) courses in the previous year (S3 Table).

### Professional identity

Only 16.9% of respondents advertised themselves exclusively as osteopaths. Respectively 82.0% and 60.4% of respondents strongly agreed with the statements ‘I strongly define myself as a healthcare practitioner’ and ‘I strongly define myself as an osteopath’ (S4 Table). Respondents strongly agreed with ‘osteopathy being regulated by law as an independent profession’ (82.3%) and considered that ‘regulation would have a positive effect on how osteopaths practice’ (69.5%). Only 25.2% of respondents strongly agreed that ‘overall, the quality of patient care provided by osteopaths in Austria is good’, and another 45% of respondents agreed with this statement. A majority of respondents strongly agreed that ‘patients should be better reimbursed for osteopathic care’ (82.3%), that ‘osteopaths in Austria would like to have better cooperation with other healthcare professionals’ (48.2%) and that osteopathy should be regulated as first line medical practice’ (56.8%). Furthermore, 51.5% strongly agreed that ‘osteopathy should develop using scientific evidence’, and another 31.1% agreed with this statement (S5 Table).

### Fees and consultation structure

Most respondents work four days a week (38.5%) and schedule 46–60 min for a new patient (68.9%) and 30–45 min for a returning patient (56.8%). The ratio of respondents that apply different fees for first and returning consultation versus respondents that do not apply different fees is 0.69, with a median fee of 81–90 euro for a first consultation and 71–80 euro for a returning patient. The median average waiting time for a first consultation was between two and four weeks and the median average number of consultations per week was 21–25. Details on the main practice characteristics are reported in Table 3.

**Table 3:** Main practice characteristics of respondents (n = 338).

**Consultation time new patient**					**Consultation time returning patient**		
Time (minutes)	n		%		Time (minutes)	n	%
<30	1		0.3		<30	10	3
30–45	52		15.4		30–45	192	56.8
46–60	233		68.9		46–60	131	38.8
>60	52		15.4		>60	5	1.5
**Fee first consultation**					**Fee following consultation**		
Fee (€)	n		%		Fee (€)	n	%
< 61	6		1.8		< 61	27	8.0
61–70	31		9.2		61–70	59	17.5
71–80	83		24.5		71–80	109	32.2
81–90	96		28.4		81–90	93	27.5
91–100	67		19.8		91–100	29	8.5
> 100	55		16.2		> 100	21	6.2
**Number of clinical working days/week**					**Number of patient consultations/week**		
Days	n		%		Patients	n	%
1	17		5.0		≤15	30	8.9
2	37		11.0		16–20	52	15.4
3	79		23.4		21–25	64	18.9
4	130		38.5		26–30	68	20.1
5	72		21.3		31–35	39	11.5
> 5	3		0.9		> 35	54	15.0
**Average waiting time for first consultation**				
Variable		n		%
Same day		1		0.3
Next working day		1		0.3
Within 2–7 working days		49		14.5
Within 8–14 working days		98		29.0
Between 2–4 weeks		106		31.4
> 1 Month		83		24.6

### Patients

While 42% of respondents declared that their patient database was evenly split between men and women, 48.7% reported that they were consulted primarily by women. According to respondents, all age groups were represented among their patients, although most were adults, with patients aged 40–65 years being consulted most frequently (63.9% reported “very often”). On the other hand, 33.7% of respondents never saw patients under the age of two. In the past year, respondents confirmed that they were consulted very often for chronic (62.1%), followed by acute (34.3%) complaints. According to respondents, patients consulted them very often for complaints of the lower spine (71.9%), neck (68.6%), upper spine (43.5%) and pelvis (42.6%). Table 4 shows the ten most specific complaints seen by respondents.

**Table 4:** The ten most common specific complaints (in descending order of ‘often’ and ‘very often’ responses).

Specific complaint	never	seldom	regularly	often	very often
Non-specific low back pain	0.3	2.4	3.3	29.6	64.5
Non-specific neck pain	0.3	1.5	4.4	26.3	67.5
Headache/ and migraines	0.3	2.7	15.7	42.3	39.1
Lumbar radiculopathy	0.9	2.7	16.3	36.7	43.5
Cervical radiculopathy	1.2	5.3	29	43.2	21.3
Cranio-mandibular complaints	2.7	16.9	39.9	26.9	13.6
Digestive disorders	3.6	17.2	41.4	28.7	9.2
Complaints during/after pregnancy	5.6	20.7	36.4	22.5	14.8
Unsettled or crying babies (colic)	36.7	15.4	13.3	13.6	21.0
Infantile postural asymmetry	18.3	24.9	25.4	16.3	15.1

Numbers in table are %

### Osteopathic skills

Only 49.7% of respondents always performed a new clinical examination at each consultation, while 34.3% confirmed doing so often. Exclusion diagnostics to determine whether or not to treat, were always performed by 66.9% of respondents, and 66.0% declared informing patients about possible risks and side effects of their recommended treatment. The most frequently used diagnostic and treatment techniques can be found, in decreasing order, in Tables 5 and 6.

**Table 5:** The most common diagnostic techniques used (in descending order of ‘often’ and ‘always’ responses).

Diagnostic technique	never	seldom	regularly	often	always	unknown
Palpation of position/ structures	0.0	0.6	1.8	8.6	88.8	0.3
Palpation of tenderness	0.0	0.6	1.8	12.4	84.9	0.3
Visual inspection	0.6	1	2.1	3.6	91.7	1.2
Palpation of movement	0.3	1.5	2.7	15.7	79.6	0.3
Assessment of visceral mobility	0.6	1.5	8.0	27.5	62.4	0.0
Assessment of the cranium	0.9	3.0	6.8	25.7	63.0	0.6
Fascial testing	1.8	6.2	12.1	32.0	47.0	0.9
Orthopaedic testing	2.1	6.2	14.8	38.2	38.5	0.3
Neurologic testing	2.7	6.5	20.1	45.0	25.4	0.3
Muscle function testing	3.0	11.2	25.2	36.4	23.7	0.6
Percussion and auscultation	13.9	19.5	29.3	23.7	10.7	3.0
Diagnostic imaging	40.8	9.2	13.3	14.8	3.9	18.1
Otoscopy	25.2	31.4	21.9	10.1	3.6	8.0
Neurolymphatic reflex tests	33.1	20.4	20.4	7.7	4.7	13.6
Blood analysis	51.8	12.1	6.5	3.0	0.6	26.0
Urine analysis	53.3	6.8	5.3	0.3	0.0	34.3

Numbers in table are %

**Table 6:** The most common therapeutic techniques used (in descending order of ‘often’ and ‘very often’ responses).

Therapeutic technique	never	seldom	regularly	often	very often	unknown
Neuro- and viscerocranial techniques	1.5	3.3	8.9	38.2	47.9	0.3
Visceral techniques	0.3	3.6	11.5	32.0	52.4	0.3
Articulatory/mobilisation techniques (GOT/TBA)	0.6	3.3	11.5	34.9	49.4	0.3
Myofascial techniques	1.2	5.3	11.0	37.6	44.1	0.9
Soft and connective tissue techniques	1.5	5.6	13.0	30.2	48.8	0.9
Functional techniques	2.7	4.4	15.7	35.5	40.8	0.9
Fluid techniques	2.4	6.5	19.8	41.4	26.9	3.0
Automatic shifting and fluid body approach	4.7	8.9	17.2	27.8	29.3	12.1
Progressive Inhibition of Neuromuscular Structures	7.4	12.4	21.0	36.1	18.3	4.7
HVLA	4.4	13.0	27.5	32.5	21.0	1.5
Muscle Energy Techniques	6.2	13.0	26.6	33.1	20.1	0.9

Numbers in table are %

GOT: General Osteopathic Treatment; TBA: Total Body Adjustment; HVLA: High Velocity Low Amplitude

Of all techniques applied to internal and sensitive areas, intraoral techniques were the most used (44.7% reported “often” to “very often”). However, techniques applied to the breasts were used to some extent (seldom to very often) by 58.9%, vaginal by 49.4% and rectal by 38.5% of respondents. Informed consent for oral techniques was requested only by 10.4% of respondents, and for genital and rectal techniques by 21.0% and 18.3% respectively. Within the recommendations given as part of the treatment plan, physical activity (53.0%) and advice on exercises (47.6%) was always discussed with patients. The reasons for referring patients to other healthcare professionals, rated as very important by respondents, were “indication of undiagnosed pathology or structural deficit” (86.7%) and “not my field of expertise” (82.8%). “Increasing patient depression/anxiety” (66.3%) was the third main reason for referring patients to another healthcare professional. Sixty per cent of respondents used supplementary methods in their osteopathic practice. “Taping/kinesiology tape” (40.2%) and “exercise therapy” (37.3%) were the most used treatment approaches, and “applied kinesiology” (16.7%) was by far the most used diagnostic approach.

## Discussion

The aim of this survey was to describe the professional profile of osteopathic practitioners working in Austria. Overall, the typical osteopathic practitioner in Austria is female, between 40 and 49 years of age, self-employed, with part-time training and an MSc-degree in osteopathy and a previous training in physiotherapy.

### Previous surveys

Prior to this study, the osteopathic profession in Austria had already been surveyed in 2003 [4] and 2011 [5]. While the 2003 survey raises questions about the validity of the questionnaire, the 2011 survey used a validated questionnaire from the Belgian Healthcare Knowledge Centre [14] adapted to the Austrian situation. This questionnaire also served as a basis for the development of the Benelux Osteosurvey [15], which in turn was the predecessor of our OPERA survey. The most important differences between these surveys have been discussed in previous publications [7, 15]. Although comparison of data is not always straightforward due to these differences in the questionnaires of the Austrian surveys, the following parts of this discussion will first focus on some possible changes over time in Austria before comparing the osteopathic profile with that of other European countries.

### Socio-demographics

Since 2003, the number of osteopaths working in Austria has increased from 192 [4], through 453 in 2011 [5] to 1,000 [3] -1,300 [personal communication between PvD and OEGO] in 2020; which is about 11 osteopaths per 100,000 inhabitants [3]. As there is no compulsory registration in Austria, these figures are only estimates.

The proportion of women among osteopaths has increased from 64% in 2003 [4] through 68.3% in 2011 [5] to 70.7% in 2020. This is in contrast with the predominantly male osteopathic profession of Belgium, Italy, Portugal and Spain [7, 9–11]. Only in Germany and Switzerland are female osteopaths in the majority with 56.7% and 54.7% respectively [16, 17]. Respondents most representative age group (40–49) is the same as those from Belgium, Germany, the UK and Switzerland [10, 16–18], which contrasts with the 30–39 age group of respondents in Italy, Portugal and Spain [7, 8, 11].

### Osteopathic education

The osteopathic profession in Austria adheres to the European CEN Standard [1] regarding its training, and only recognises part-time education with a minimum of 1,500 hours within five to six years. There are two private osteopathic education institutes (OEI) in Austria. One OEI [19] provides a four year part-time basic course and then cooperates with an Austrian university to deliver a degree as an academic expert (osteopathy) (60 ECTS) or a master’s degree (120 ECTS). Both degrees have access to the profession. The other OEI [20] cooperates with a UK university and delivers a master’s degree after a four year part-time training program. The DO brand, which was the second most common highest qualification and is awarded by the profession itself, is the subject of evolution in Europe and is steadily being replaced by academic degrees such as a bachelor’s and a master’s degree in osteopathy as a result of a gradual regularisation and academic development of the profession. However, the survey responses showed no relation between the degrees and the year of graduation. At least in Austria, the replacement of the DO brand by academic degrees is not clear. This can be due to the fact that regularisation of osteopathy is still non-existent and/or that the academisation is not progressing steadily, or that there are still other factors that may play a role in maintaining the DO brand (see “academic expert degree” further).

Due to the fact that osteopathy in Austria is not recognised, it falls under the Medical Act, meaning that only physicians and physiotherapists are allowed to practice osteopathy, whereby physiotherapists are only allowed to work with the referral of a physician [21]. This explains why almost all respondents had preliminary healthcare training, which is in agreement with respondents from Belgium, Spain and Germany where the vast majority had prior training as a physiotherapist [7, 10, 16]. On the other hand, almost one third of respondents of Belgium, Italy and Portugal [7, 9–11] received a full-time education, which contrasts the two and six percent of Austria and Germany respectively [16].

Curiously, one quarter of respondents could not answer which osteopathic qualification they held. This might have to do with the previously mentioned “academic expert (osteopathy)” degree which was not an option in the questionnaire as this was overlooked in the pilot process. Only OPERA Spain came up with an almost similar result, but most likely for different reasons. Reasons that may also have played a role in the present survey, such as respondents who did not complete their training and still presented themselves as osteopaths, as the profession is not regulated and therefore not protected. Given the differences among Austrian osteopathic qualifications, it would be necessary to continue the process of regulation in order to promote better professional homogeneity and patient care.

### Osteopathic identity

Although a majority of respondents strongly agreed to define themselves as osteopaths and felt that being an osteopath was important to them, only 17% advertised themselves exclusively as osteopaths, which is by far the lowest percentage of all European countries that dealt with this topic, as far as we know [7, 8, 10, 11, 16]. Thus, it seems that although respondents perceived a strong osteopathic identity, they failed to fully make themselves known to the public as osteopaths. A possible explanation is that respondents do not feel comfortable presenting themselves as representatives of a profession that is not yet recognised, let alone regulated.

While there is substantial agreement that "Osteopathy in Austria should be regulated as a first-line medical practice”, it is unclear whether all respondents are fully aware of the implications for the profession. It may be that most respondents only associate this statement with the "primary contact" feature from the CEN Standard [1]. Other features, such as providing integrated, accessible health care services by clinicians accountable for addressing a large proportion of personal health care needs, developing a sustained partnership with patients, and practising in the context of family and community, may be overlooked in this process. They certainly have solid implications for osteopathic training and the inclusion of osteopathic care as a mainstream healthcare component.

### Practice characteristics

By analogy with previous surveys, respondents of this survey were most likely to work alone and have consultations that are likely to last 46–60 minutes for a first-time patient and 30–45 minutes for a returning patient. However, the average number of patients consultations per week decreased from 33 in 2003 [4] through 31.2 in 2011 [5] to 21–25 in 2020. Only Portugal had a similar number of consultations per week; all other European countries surveyed had numbers around 30 [7, 9–11, 16].

Whereas 46% of respondents in the current study work in a team, in 2011 this was 67% [5]. This goes against the trend demonstrated by [9] whereby the new osteopathic generation works as an interprofessional team with other healthcare professionals, recognising the added value that interprofessional care brings to their patients. The authors demonstrated this by osteopaths working in teams being significantly younger than their colleagues working as sole practitioners. The most common team members have not changed over time and are still physiotherapists and other osteopaths, which is also the case in several other European countries [7, 9, 10, 16].

### Patient profile

Although the current survey was practitioner-oriented, the most common patient reported by respondents was female, aged between 40 and 65, with chronic complaints localised to the lower spine or neck. This profile is very similar to that of patient-oriented studies showing that the majority of patients are adults with mean and mode age ranges between 40 and 65 years and were more likely to be women than men. They consult an osteopathic practitioner with musculoskeletal complaints mostly in the cervical and lumbar region [17, 22–26].

### Osteopathic skills

The frequency of use of osteopathic techniques is only partially comparable because of the different surveys used in Austria over time. Although the comparison of a four- and five-point Likert scale is difficult, it can be argued that the use of HVLA techniques in Austria, which were regularly (four-point Likert scale of never-rare-occasionally-regularly) used by a similar 49% and 49.6% in 2003 and 2011 respectively, dropped to 21% of “very often” use in the present survey. Two of the three most commonly used techniques by respondents in the present survey (i.e. visceral and cranial techniques), were techniques that were regularly used by 93% and 91% of respondents in 2003, and by 61% and 78% in 2011.

For the elaboration of a working diagnosis, respondents mainly used palpation skills for diagnostic techniques, which is in accordance with other European studies [7, 9–11, 16]. The frequent use of visceral therapeutic techniques is in accordance with the results of other European countries [7, 9, 10, 16, 17], but contrasts to that of UK osteopaths, of which 32% of respondents stated to never use visceral techniques [18] and more recent retrospective consultation data shows that visceral techniques are only applied in 5.1% during a first and 2.8% during a second consultation [24].

Although intraoral techniques were the most commonly used techniques for internal and sensitive areas, others (vaginal, rectal, breast), although used to a lesser extent, were used by over one-third to one-half of the respondents. A similar use of these diagnostic and therapeutic techniques was also observed in other European countries [7, 10, 11, 16]. Only in Italy was the use considerably lower [9].

The very low rates of respondents requiring written consent before the use of techniques in internal or sensitive areas is very surprising. It is all the more surprising because the vast majority of respondents already have a medical degree and have thus been familiarised through their previous training with the rules of written consent within the framework of good clinical practice. It seems that within osteopathic training there is still a need to address this issue. The UK Osteopathic Practice Standards [27] require an osteopath to have their patient’s valid consent before they examine or treat the patient, and several professional associations in different European countries, including OEGO, urge their members to ask for a written consent when diagnosing or treating internal or sensitive areas [28, 29].

### Strength and limitations

Although osteopathic practitioners in Austria had been surveyed in the past, this updated survey gives supplementary information, especially with regard to the professional identity of respondents and their views on the profession. However, some limitations should be considered when interpreting the results of this survey. Although the osteopathic profession seems to be fairly well organized in Austria and there is only one single interest group for the profession, the sample size may be biased due to the fact that there is no mandatory register of osteopaths to provide data. Also, because practitioners were responsible for data entry, the results may have been influenced by respondent bias.

## Conclusions

Although the osteopathic profession has already been surveyed in 2003 and 2011, the OPERA-AU study represents the first published document to determine the characteristics of osteopathic practitioners in Austria using an extensive, national sample. It provides a comprehensive dataset profiling osteopathic practitioners and their practice characteristics. Our findings could have implications for the development of the profession. First, it represents the most informative document related to the osteopathic community in Austria, bringing new information on where and how osteopathy is practised, and how osteopaths profile themselves. Secondly, comparisons between countries were discussed in order to highlight differences across European countries. Finally, the information provided may contribute to the evidence used by stakeholders and policy makers for the future regulation of the profession in Austria.

## Supporting information

S1 Fig Team members working with osteopaths. (TIF)

S1 Table Geographical distribution by federal state and membership in a professional osteopathic association (n = 338). (DOCX)

S2 Table Other professional activities. (DOCX)

S3 Table Osteopathic training and life-long learning characteristics (n = 338). (DOCX)

S4 Table Osteopath identity statements. (DOCX)

S5 Table Views as an osteopath statements. (DOCX)
