# Supplementary figures and images for: The Austrian Osteopathic Practitioners Estimates and RAtes (OPERA): A cross-sectional survey
Source: PLoS One. 2022 Nov 28;17(11):e0278041. doi: 10.1371/journal.pone.0278041 (PMC9704649; doi:10.1371/journal.pone.0278041)

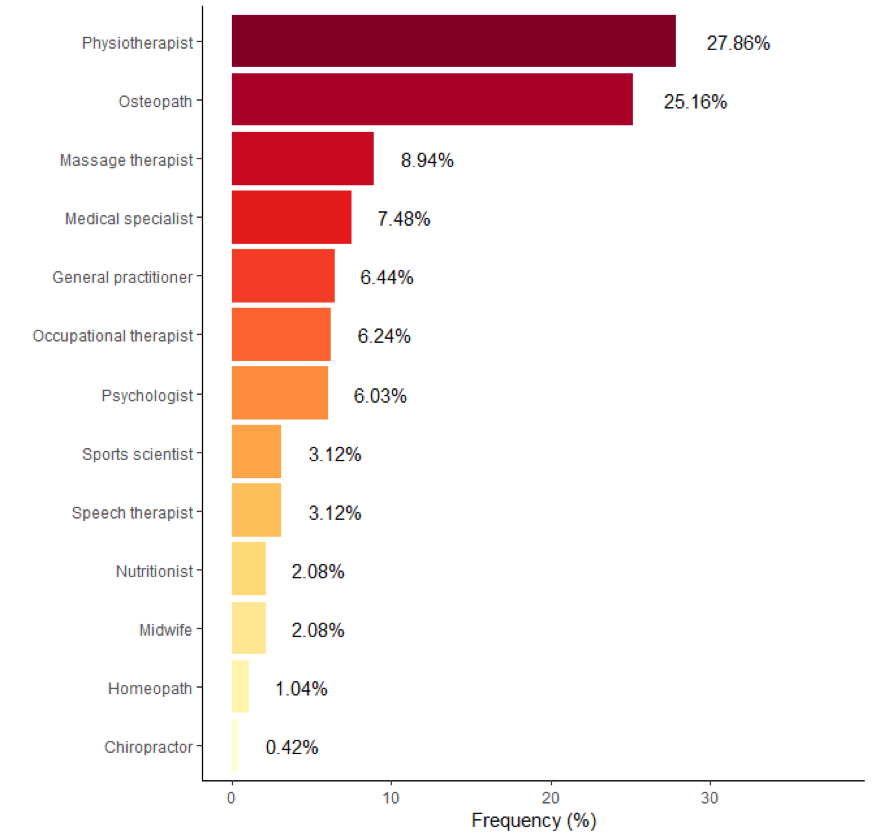

Supplement: S1 Fig — (TIF) [file pone.0278041.s001.tif]
