# Supplementary material for: The Austrian Osteopathic Practitioners Estimates and RAtes (OPERA): A cross-sectional survey
Source: PLoS One. 2022 Nov 28;17(11):e0278041. doi: 10.1371/journal.pone.0278041 (PMC9704649; doi:10.1371/journal.pone.0278041)
Supplement: S1 Table — (DOCX) [file pone.0278041.s002.docx]

**S1 Table. Geographical distribution by federal state and membership in a professional osteopathic association (n=338).**

| **Variable** | **n** | **%** |
| --- | --- | --- |
| **Geographical distribution by federal state** | | |
| Burgerland | 4 | 1.2 |
| Kärnten | 18 | 5.3 |
| Niederösterreich | 52 | 15.4 |
| Oberösterreich | 52 | 15.4 |
| Salzburg | 31 | 9.2 |
| Steiermark | 48 | 14.2 |
| Tirol | 31 | 9.2 |
| Vorarlberg | 14 | 4.1 |
| Wien | 88 | 26.0 |
| **Professional osteopathic association** | | |
| Yes | 252 | 74.6 |
| No | 86 | 25.4 |
| **Association** | | |
| OEGO | 247 | 81.2 |
| Other in Austria | 49 | 16.1 |
| Other abroad | 8 | 2.6 |
