# Supplementary material for: The Austrian Osteopathic Practitioners Estimates and RAtes (OPERA): A cross-sectional survey
Source: PLoS One. 2022 Nov 28;17(11):e0278041. doi: 10.1371/journal.pone.0278041 (PMC9704649; doi:10.1371/journal.pone.0278041)
Supplement: S2 Table — (DOCX) [file pone.0278041.s003.docx]

**S2 Table.** **Other professional activities.**

| **Activity** | **n** | **%** |
| --- | --- | --- |
| clinical physician | 22 | 9.0 |
| clinical physiotherapist | 126 | 51.2 |
| other healthcare practitioner | 9 | 4.0 |
| lecturing osteopathic courses in learning | 27 | 11.0 |
| supervising osteopathic clinical trainees | 26 | 11.0 |
| supervising osteopathic research dissertations | 5 | 2.0 |
| researchers | 0 | 0.0 |
| work for a professional osteopathic regulatory body or association | 7 | 2.9 |
| other professional activities | 24 | 9.8 |
