# Supplementary material for: The Austrian Osteopathic Practitioners Estimates and RAtes (OPERA): A cross-sectional survey
Source: PLoS One. 2022 Nov 28;17(11):e0278041. doi: 10.1371/journal.pone.0278041 (PMC9704649; doi:10.1371/journal.pone.0278041)
Supplement: S3 Table — (DOCX) [file pone.0278041.s004.docx]

**S3 Table. Osteopathic training and life-long learning characteristics (n= 338).**

| **Descriptor** | **Variable** | **n** | **%** |
| --- | --- | --- | --- |
| **Type of training** | Part-time | 331 | 97.9 |
|  | Full-time | 7 | 2.1 |
| **Duration of the training** | 3 years | 1 | 0.3 |
|  | 4 years | 5 | 1.5 |
|  | 5 years | 74 | 21.9 |
|  | 6 years | 97 | 28.7 |
|  | >6 years | 161 | 47.6 |
| **Type of osteopathic academic**  **qualification** | Diploma Osteopathy (DO) | 113 | 33.4 |
|  | Bachelor (Graduate) | 6 | 1.8 |
|  | Master | 131 | 38.8 |
|  | PhD | 0 | 0.0 |
| **Continuous Professional**  **Development (CPD)** | Yes | 302 | 89.4 |
|  | No | 36 | 10.7 |
| **Previous qualification(s)** | Massage therapist | 31 | 7.4 |
|  | Medical doctor | 23 | 5.5 |
|  | Nurse | 4 | 1.0 |
|  | Physiotherapist | 301 | 71.5 |
|  | Chiropractor | 1 | 0.2 |
|  | Sport scientist | 10 | 2.4 |
|  | Other healthcare training | 16 | 8.1 |
|  | No prior training | 1 | 0.2 |
