# Supplementary material for: The Austrian Osteopathic Practitioners Estimates and RAtes (OPERA): A cross-sectional survey
Source: PLoS One. 2022 Nov 28;17(11):e0278041. doi: 10.1371/journal.pone.0278041 (PMC9704649; doi:10.1371/journal.pone.0278041)
Supplement: S4 Table — (DOCX) [file pone.0278041.s005.docx]

**S4 Table. Osteopath identity statements.**

| **Statement** | **strongly**  **disagree** | **disagree** | **neither agree or disagree** | **agree** | **strongly agree** |
| --- | --- | --- | --- | --- | --- |
| I strongly define myself as an osteopath. | 1.2 (4) | 0.9 (3) | 11.5 (39) | 26.0 (88) | 60.4 (204) |
| Being an osteopath is important to me. | 0.9 (3) | 2.4 (8) | 7.7 (26) | 24.0 (81) | 65.1 (220) |
| I’m proud to be an osteopath. | 0.6 (2) | 2.1 (7) | 5.9 (20) | 18.6 (63) | 72.8 (246) |
| I strongly define myself as a health care practitioner. | 0.3 (1) | 0.9 (3) | 3.9 (13) | 13.0 (44) | 82.0 (277) |
| Being a health care practitioner is important to me. | 0.6 (2) | 0.3 (1) | 10.7 (36) | 19.8 (67) | 68.7 (232) |
| Numbers in table are % (n) | | | | | |
