# Supplementary material for: The Austrian Osteopathic Practitioners Estimates and RAtes (OPERA): A cross-sectional survey
Source: PLoS One. 2022 Nov 28;17(11):e0278041. doi: 10.1371/journal.pone.0278041 (PMC9704649; doi:10.1371/journal.pone.0278041)
Supplement: S5 Table — (DOCX) [file pone.0278041.s006.docx]

**S5 Table. Views as an osteopath statements.**

| **Statement** | **strongly**  **disagree** | **disagree** | **neither agree or disagree** | **agree** | **strongly agree** |
| --- | --- | --- | --- | --- | --- |
| Medical professionals (physicians, physiotherapists, etc.) in Austria see osteopathy as a distinct healthcare discipline. | 12.4  (42) | 25.7  (87) | 34.3  (116) | 21.6  (73) | 5.9  (20) |
| Overall the quality of patient care provided by osteopaths in Austria is good. | 1.5  (5) | 3.9  (13) | 24.6  (83) | 45.0  (152) | 25.2  (85) |
| Osteopathy should be regulated by law as an independent profession in Austria. | 0.9  (3) | 1.5  (5) | 5.3  (18) | 10.1  (34) | 82.3  (278) |
| Regulation would have a positive effect on how osteopaths practice. | 1.2  (4) | 2.4  (8) | 10.7  (36) | 16.3  (55) | 69.5  (235) |
| Patients should be better reimbursed for osteopathic care in Austria. | 0.9  (3) | 0.9  (3) | 6.2  (21) | 9.8  (33) | 82.3  (278) |
| Medical professionals (physicians, physiotherapists, etc.) in Austria see osteopathy as a subgroup of reimbursable manual therapy. | 6.2  (21) | 17.5  (59) | 37.0  (125) | 27.8  (94) | 11.5  (39) |
| Osteopaths in Austria would like to have better cooperation with other healthcare professionals. | 0.3  (1) | 0.9  (3) | 16.3  (55) | 34.3  (116) | 48.2  (163) |
| Osteopathy in Austria should be regulated as a first line medical practice. | 3.0  (10) | 4.7  (16) | 14.2  (48) | 21.3  (72) | 56.8  (192) |
| Osteopathy in Austria should be regulated as an allied health profession (e.g. physiotherapy or occupational therapy). | 41.7  (141) | 22.8  (77) | 18.3  (62) | 10.4  (35) | 6.8  (23) |
| Osteopathic practice should develop using contemporary scientific evidence. | 0.6  (2) | 3.3  (11) | 13.6  (46) | 31.1  (105) | 51.5  (174) |
| Osteopathic principles should be re-defined and amended in line with new knowledge. | 6.2  (21) | 8.9  (30) | 27.2  (92) | 32.3  (109) | 25.4  (86) |
| Numbers in table are % (n). | | | | | |
